# Reconstructing the Mexican Tropical Dry Forests via an Autoecological Niche Approach: Reconsidering the Ecosystem Boundaries

**DOI:** 10.1371/journal.pone.0150932

**Published:** 2016-03-11

**Authors:** David A. Prieto-Torres, Octavio R. Rojas-Soto

**Affiliations:** Red de Biología Evolutiva, Laboratorio de Bioclimatología, Instituto de Ecología, A.C., Xalapa, Veracruz, México; Universidade Federal de Goiás, BRAZIL

## Abstract

We used Ecological Niche Modeling (ENM) of individual species of two taxonomic groups (plants and birds) in order to reconstruct the climatic distribution of Tropical Dry Forests (TDFs) in Mexico and to analyze their boundaries with other terrestrial ecosystems. The reconstruction for TDFs’ distribution was analyzed considering the prediction and omission errors based upon the combination of species, obtained from the overlap of individual models (only plants, only birds, and all species combined). Two verifications were used: a primary vegetation map and 100 independent TDFs localities. We performed a Principal Component (PCA) and Discriminant Analysis (DA) to evaluate the variation in the environmental variables and ecological overlap among ecosystems. The modeling strategies showed differences in the ecological patterns and prediction areas, where the “all species combined” model (with a threshold of ≥10 species) was the best strategy to use in the TDFs reconstruction. We observed a concordance of 78% with the primary vegetation map and a prediction of 98% of independent locality records. Although PCA and DA tests explained 75.78% and 97.9% of variance observed, respectively, we observed an important overlap among the TDFs with other adjacent ecosystems, confirming the existence of transition zones among them. We successfully modeled the distribution of Mexican TDFs using a number of bioclimatic variables and co-distributed species. This autoecological niche approach suggests the necessity of rethinking the delimitations of ecosystems based on the recognition of transition zones among them in order to understand the real nature of communities and association patterns of species.

## Introduction

The development of more rigorous approaches for evaluating and understanding biodiversity distribution patterns is critical due to current trends in ecosystem perturbations [1–3]. Considerable conservation efforts have recently focused on predicting the geographical distributions of species independently of one another [4–6]. However, given the limited time and availability of resources for conserving and managing biodiversity based on a species-by-species approach, our ability to meet the challenges of biodiversity conservation hinges on the successful conservation of entire ecosystems in order to maintain the integrity of ecological processes [1–3].

The definition and delimitation of communities has been generally approached from the perspectives of physiognomy (i.e. plant composition and abundance) and ecological interactions, in addition to a consideration of the history and evolution of the taxonomic groups involved [7–10]. Nevertheless, the challenge of modeling entire communities, as well as ecosystems, remains controversial and more research is needed to understand the traits and factors that are important in defining the geographic and ecological distribution patterns of species involved [5,11,12].

Ecological Niche Modeling (ENM) has increasingly been used to predict ecosystems distributions over time–considering the effects of climate change–based on mapping the location of climatically suitable areas [3,13–15]. Recently, there have been attempts to forecast entire ecosystems distributions based on randomly chosen training points in a defined area of pre-established boundaries within vegetation communities, which are used for modeling performance (hereafter “single-ecosystem models"; e.g. Werneck *et al*. [16,17]; Ponce-Reyes *et al*. [14]). However, these attempts might produce misleading results due to the mismatch between species distribution and pre-established ecosystem boundaries; which are usually defined by the dominant species that possibly also occur in others ecosystems. In addition, the strategy employed in a single-ecosystem model contrasts with one of the foundations of niche modeling–species show individual responses to climate variation [3,13–15].

Considering that species and ecosystems as well as their spatial configuration throughout their distribution are not static in the time, the community-based species distribution modeling or SDM represents an alternative approach to the statistical analysis of species co-occurrence in environmental space, which may help define biological communities [5,15,18,19]. Community-based modeling combines distributions from several species to produce synthetic representations of the spatial pattern of biodiversity distribution at a collective level [11,20]. This approach implicitly assumes that statistical patterns of co-occurrence among species potentially capture meaningful biotic interactions among species that may play an important role in shaping species distributions [6,12,21], thereby also providing a useful tool for modeling community and ecosystem dynamics [3,15,20].

The distributional area of species is a complex expression of factors, including ecology and evolutionary history [19,22]. Soberón and Peterson [19] illustrate by the BAM diagram that three factors are particularly important, and assumed that a species will be present at a given point (i.e. distribution area [Go]) when these three conditions are met: (i) abiotic conditions (or “A”) are favorable; (ii) both interactions positive or negative (or “B”) with other species are necessary to maintain populations; and (iii) the species will be present only in a region (defined as “M”) that is reachable from established distributional areas in ecological time (i.e., dispersal limitations). However, these factors interact dynamically, with differing degrees of strength and at different scales, to produce the complex and fluid entities that are defined as potential distribution areas (see Soberón and Peterson [19] for a complete discussion). It may also be noted that the invasive areas (or Gi) are defined as the geographic expression of the species’ niches [19,23], where conditions (abiotic and biotic) are suitable for positive population growth but are not accessible to these species.

Based on these implications and considering the community modeling strategy, we propose that instead of SDM, the ecological niche models (ENM)—including both Go and Gi areas [19]—of multiple species and taxonomic groups can be used to analyze the collective properties of species requirements as an approach to reconstruct ecosystems, assuming that species share similar ecological requirements, particularly those that are ecologically restricted [11,12,20,21]. This new idea implies, to some extent, potential niche similarity among species belonging to diverse taxonomic groups based on their evolutionary convergence [5,13,15], thus, we can assume that it is not necessary to analyze all occurring species to reconstruct the ecosystems (e.g. Prieto-Torres *et al*. [3], Collevatti *et al*. [13], and Rojas-Soto *et al*. [15]). However, here arise fundamental questions of how much of such similarity (overlap) and how many species should be necessary to recognize the geographic and ecological distributions of ecosystems, as well as the transitional areas among them.

To address these needs, we focus on a community-level modeling strategy to analyze how the potential ecological distributions of species help to reconstruct the distribution of endangered Tropical Dry Forests (TDFs) in Mexico. Specifically, we analyzed whether predictions made with an accumulative model (i.e. sums of the ENM of different species based on three strategies of taxonomic model combinations) resulted in differences for the reconstruction of Mexican TDFs, and finally we contrasted them with a single-ecosystem model obtained from a TDFs map for Mexico [24]. This information is of great value because depending on the performance of the modeling approach, future analyses may confer in the development of conservation strategies and in the definition of potential geographic and ecological distributions of ecosystems across distinct spatial-temporal scales, for example, by measuring the potential effect of future scenarios of climate change, or by contributing towards the understanding of the historical dynamic of ecosystems [3,13,15].

## Materials and Methods

### Study area

The New World TDFs are distributed discontinuously from northwestern Mexico to northern Argentina and southwestern Brazil in disjunct areas of varying size [25–27]. Their geographic variants have not always been classified within the same vegetation type or associated with the same biogeographic unit. However, recent studies have attempted to define the distribution and phytogeography of the TDFs as a natural ecosystem widely distributed throughout the Neotropics [25–30]. For this study, TDFs is broadly defined as an ecosystem typically dominated by deciduous trees (> 50%), existing in regions with a mean annual temperature >25°C, a total annual precipitation between 700 and 2,000 mm, and with the presence of three or more dry months every year [25–27,29–31]. The forests are mostly deciduous during the dry season, and the degree of deciduousness increases with declining rainfall, although the driest TDFs have more evergreen and succulent species. Typically, trees in TDFs have a smaller height and lower basal area than those from tropical rain forests. Thorny species are often prominent, and it is essentially a tree-dominated ecosystem with a more or less continuous canopy, where grasses are minor elements [27,31,32].

**Fig 1:**
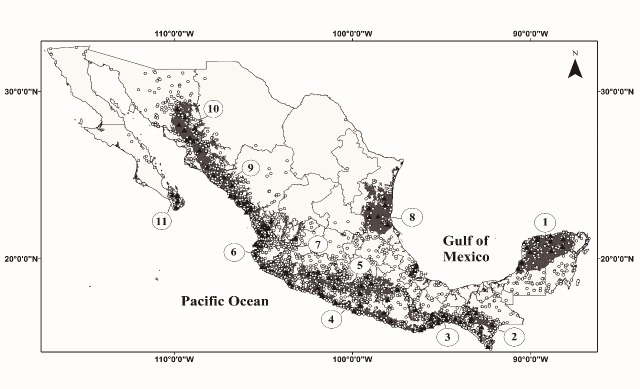
Unique locality records in Mexico used for species model performance (indicated by white dots). Primary TDFs (grey shading) and known localities of TDFs (indicated by black triangles) in Mexico were used to evaluate ENMs of species for the TDFs reconstruction. Numbers correspond to areas: Yucatan forests (1), Chiapas forests (2), Centro American Pacific forests (3), Pacific South forests (4), Balsas river basin forests (5), Jalisco forest (6), Bajío forests (7), Tamaulipas-Veracruz forests (8), Sinaloa forests (9), Sonora and Sinaloa forests (10), and Cape forests (11). The figure was adapted from INEGI’ map (2003).

In Mexico, the available information confirms that TDFs (including the “*Selvas bajas caducifolias y subcaducifolias*”, “*Selvas medianas caducifolias y subcaducifolias*”, and “*Selvas Espinosas*” as classified in Spanish) are found along the Pacific coast forming a nearly continuous strip, from Sonora and the Cabo Region down to the border with Gautemala (including Jalisco, and in the Santiago and Balsas river basins; Fig 1). On the Gulf coast, TDFs are present in more isolated and discontinuous areas throughout southern Tamaulipas, central Veracruz, and the Yucatan Peninsula [24,27,33–38]. The spatial distribution and appearance of these forests are composed of a heterogeneous matrix of topographic, climatic, and edaphic conditions [36–39]. Accordingly, TDFs in Mexico exhibit considerable spatial variation in structure and species composition. Thus, we define TDFs by the presence of deciduous trees, the general physiognomy described above, and their climatic affinity [27,29,39].

### Species and occurrence records

We used two taxonomic groups, plants and birds, since: 1) plants species are most frequently used to define vegetation types, which are linked to ecological conditions that represent ecosystems and determine the distributions of entire communities [7–10,40]; and 2) birds are the group with the largest amount of occurrence information for the implementation of ENMs, and thus we have a general understanding of their biogeography [41], in comparison to other animal groups.

We analyzed 30 species—15 birds and 15 plants (Table 1)—considered to be ecologically associated or dominant species of Mexican TDFs [25,36,37,40,41]. While this species number may appear as arbitrary, the value was established based on previous studies (including preliminary analyses with TDFs) indicating that the use of 20 to 30 species, is sufficient to obtain validated results in the ecosystems reconstructions [3,13,15]. Is important to note that within the selected groups, we included 11 species that are widely distributed in the Mexican TDFs and with partial distributions in other ecosystems (such as Temperate Forests), whereas others (19 species) are restricted or endemic to TDFs (Table 1). All selected species have relatively high number of occurrences records. Species with two contrasting life forms were included in the case of plants (i.e., trees *vs*. shrubs) and different trophic guilds (e.g., granivorous or insectivorous) in the case of birds. Also, for both groups, we selected species with dissimilar geographical distributions patterns (Table 1): those endemics to Mexico or Mesoamerica, those possessing a larger Neotropical distribution, and species that are phylogenetically unrelated [3].

**Table 1:** Species modeled and used for Tropical Dry Forests reconstruction in Mexico. Families and species were assigned according to: IOC World Bird List (Gill & Donsker, 2015), APG III (APG, 2009), and The Plant List (2013).

Species	Records	Habits/life form	Distribution
BIRDS			
TROCHILIDAE			
*Amazilia rutile*	1082	Nectarivorous	Mesoamerica*
*Heliomaster constantii*	460	Nectarivorous	Mesoamerica
CAPRIMULGIDAE			
*Antrostomus ridgwayi*	106	Insectivorous	Mesoamerica*
*Nyctiphrynus mcleodii*	30	Insectivorous	Mexico
CRACIDAE			
*Ortalis poliocephala*	521	Frugivorous	Mexico*
CORVIDAE			
*Calocitta colliei*	531	Frugivorous- Insectivorous	Mexico*
PASSERELLIDAE			
*Peucaea humeralis*	243	Granivorous	Mexico
*P*. *ruficauda*	966	Granivorous	Mesoamerica*
POLIOPTILIDAE			
*Polioptila albiloris*	576	Insectivorous	Mesoamerica*
*P*. *nigriceps*	280	Insectivorous	Mexico
TYRANNIDAE			
*Deltarhynchus flammulatus*	48	Insectivorous	Mexico*
PICIDAE			
*Melanerpes chrysogenys*	1092	Insectivorous	Mexico*
PSITTACIDAE			
*Eupsittula canicularis*	924	Frugivorous-Granivorous	Mesoamerica
STRIGIDAE			
*Megascops guatemalae*	209	Carnivores	Neotropical
*M*. *seductus*	44	Carnivores	Mexico*
**PLANTS**			
ANACARDIACEAE			
*Amphipterygium adstringens* (Schltdl.) Standl.	103	Tree	Mexico*
BIXACEAE			
*Cochlospermum vitifolium* (Willd.) Spreng.	130	Tree	Mexico
BURSERACEAE			
*Bursera fagaroides* (Kunth) Engl.	255	Shrub	Mexico
CONCOLCULACEAE			
*Ipomoea wolcottiana* Rose	28	Tree	Mexico*
EUPHORBIACEAE			
*Jatropha cordata* (Ortega) Müll.Arg.	65	Shrub	Mexico*
FABACEAE			
*Acacia cochliacantha* (Willd.)	277	Shrub	Mexico*
*Enterolobium cyclocarpum* (Jacq.) Griseb.	109	Tree	Neotropical*
*Haematoxylum brasiletto* H.Karst.	112	Tree	Mesoamerica*
*Lysiloma divaricatum* (Jacq.) J.C. Macbr.	249	Tree	Mesoamerica
*L*. *watsonii* Rose	25	Shrub	Mexico*
*Senna atomaria* (L.) H.S. Irwin & Barneby	304	Tree	Mesoamerica*
MELIACEAE			
*Swietenia humilis* Zucc.	45	Tree	Mesoamerica
*Trichilia hirta* L.	119	Tree	Neotropical*
RHAMNACEAE			
*Ziziphus amole* (Sessé & Moc.) M.C. Johnst	106	Shrub	Mexico
RUTACEAE			
*Zanthoxylum fagara* (L.) Sarg.	108	Tree	Neotropical*

* Species ecological and geographically restricted (i.e. endemic) for Tropical Dry Forests.

A database of available records per species was gathered from collection records from the Global Biodiversity Information Facility database (GBIF; www.gbif.org), the Ornithological Information System (ORNIS, www.ornisnet.org), and the Atlas of the Birds of Mexico [42]. For birds, some records were obtained directly from fieldwork. Records repeated in multiple sources were removed and only unique information was used; all doubtful and ambiguous localities (i.e. information that could not be verified) were omitted. Throughout this process, a total of 9,244 unique occurrence data points (i.e. geographic localities where the species are known to occur) were assembled for species in the two broad taxonomic groups (Table 1). Geographic coordinates were used in decimal degrees based on the WGS84 datum.

Additionally, in order to test the performance of a multi-species modeling approach, we generated a single-ecosystem model of Mexican TDFs using the maximum entropy algorithm (see below) and 500 presence points randomly selected from a pre-established TDFs map [24].

### Ecological Niche Modeling

We performed ENMs (for both multi-species modeling and single-ecosystem approaches) using MaxEnt version 3.3.3k [4,43,44], which considers the principle of maximum entropy to calculate the most likely distribution for each species considering two data inputs: occurrence localities and digital layers of environmental conditions [43,44]. Given that configuration spatial extent of training is a prerequisite to effective model implementations [45,46], the individual models were calibrated according the accessibility area of each species (or "M" sensu BAM diagram [19,47]), and then were projected to the extension of Mexico. The accessibility areas for each species was estimated using a geographical clipping based on the classification of terrestrial ecoregions [48], the Biogeographical Provinces [49,50], and altitude range limits; which represents the species’ historical barriers to dispersal [51,52]. In other hand, to characterize the ecological niches, we used 30” resolution (~1 km^2^ cell size) interpolated climate data from the WorldClim project, which included a set of 19 climatic variables that summarize aspects of precipitation and temperature [53].

The procedures for ENM using the 19 environmental variables have been discussed extensively elsewhere [46,47,54–56], including the fact that correlations between climate variables may exist, as well as some methods to solve it (e.g. use the scores of Principal Component Analysis (PCA) as variables [57,58]). However, despite that good principles of modeling suggest to use less variables, we have not eliminated any of 19 variables in order to simplify the comparisons among the model results (including the effects or importance of each variable for the species [see S1 Table]); especially for the following reasons: (1) we used different calibration extensions areas for each species during the modeling process, which included areas inside and outside of country, depending if the species possessed larger distributions (i.e. Mesoamerican or Neotropical); and (2) we selected both type of species, i.e. those restricted to TDFs and those with partial distributions into other ecosystems. These points suggest, to some extent, that variables interact differently depending on the particular conditions of the unique and local conditions (e.g. see Trejo [38, 39]) used as areas for modeling, which are not the same for all species [45,46,58]. Accordingly, alternative methods–as PCA to model species (for cite a case)–result in multiple and complex analysis, which make difficult the biological interpretation of data obtained in this study. Nevertheless, in our previous analyses (results not shown) using less variables–as suggests some authors [47,54]–and the PC scores as predictions variables [57,58], the predicted distribution patterns were geographically similar to those obtained with 19 variables, but with best performance values for this last strategy (additional information can be requested to the corresponding authors).

Models were generated using a random sampling of 70% of the localities as training data and the remaining 30% as testing data. In addition, we ran 1,000 iterations with no extrapolation in order to avoid artificial extrapolations from extreme values of the ecological variables; as such parameters are biased towards the environmental envelope of background points and occurrence data [43,44]. All other parameters in MaxEnt were maintained at default settings. We used the logistic response to obtain the values for climatic suitability (continuous probability from 0 to 1), which were subsequently converted into binary presence-absence values based on established threshold value.

Various methods to determine model thresholds exist, as its selection commonly depends on the dataset used or the objectives of the model, and varying from species to species (e.g. Liu *et al*. [59,60], Pearson *et al*. [61], and Jiménez-Valverde and Lobo [62]). For our purposes, we used a threshold defined as the “fixed omission value 5 (or FOV5)” for training data, which can be ecologically interpreted as the identification of pixels predicted to be at least as suitable as pixels where species presence has been previously recorded, rejecting only 5% of training presence records [3,61,63]. This threshold gives greater value to presence records than the background points generated by MaxEnt, which allows us to minimize, to an extent, commission errors (or over-predictions) in the binary maps [3,63].

Finally, we evaluated the performance of the model by calculating the values of commission and omission errors, the Area Under the Curve (AUC) of the Receiver Operating Characteristic (ROC) curve [43,44], as well by applying the partial ROC curves test. This later criterion is used to solve problems associated with the AUC, avoiding an inappropriate weighing of the omission and commission components of the analysis [47,64,65]. We calculated partial AUCs using the Tool for Partial-ROC V. 1.0. [66] with 30% of the unique occurrence data points for independent model evaluation and presented the partial ROC results as the ratio of the AUC model to the null expectation ("AUC ratio"). Bootstrapping manipulations to permit evaluation of statistical significance of AUCs when compared with null expectations were achieved by resampling 50% of the points, replacing values 1,000 times from the overall pool of data. Significance (e.g. elevation above the line of null expectation) was assessed by ranking the observed value (calculated AUC ratio) with the values from pseudo-replicates, following the proposal of Peterson et al.[65].

### Reconstruction of TDFs based on autoecological niche approach

To reconstruct potential TDFs based on this approach of multi-species modeling, distribution maps (hereafter ‘‘modeled TDFs”) were obtained by overlapping of individual species predictions maps. Because there are diverse degrees of disturbance and human-related modification of the natural environment that are not considered by the ENMs, our modeled TDFs maps was compared with the map of the natural vegetation (hereafter ‘‘primary TDFs map”; Fig 1) generated by Instituto Nacional de Estadística, Geografía e Informática (INEGI), which describes the potential vegetation of a given area without considering human-related modifications [24]. The primary TDFs map was stored in an ASCII ‘‘raster” format and imported to ArcMap 10.2 [67], with the same pixel size of the environmental layers (~1 km^2^). The study area reached an extent of ~400,000.00 km^2^. This comparison among maps (including the single-ecosystem model) allowed us to assess the predicting of TDFs areas in terms of omission (underprediction) and commission (overprediction; [3,15]), and at the same time, to discuss the distribution boundaries among officially recognized vegetation types [24,34,36,37].

We evaluated the efficiency of ENMs in predicting the geographic distribution of TDFs using three approaches: (1) by comparing the predicted distribution of each species in relation to the primary TDFs map; (2) by considering the sums of species models (from a range of 1 to 30 models) and their correspondence with the primary TDFs map; and (3) by comparing the distribution of the modeled TDFs with 100 TDFs localities that were independently verified and obtained from collections, herbariums, specialized literature, and field work (Fig 1; S2 Table). For this last approach, the coincidence between the predicted TDFs through modeling and the independently verified TDFs localities was represented in an two-dimensional ecological space using the two most important environmental variables out of 19 climatic variables, defined by a principal component analysis (PCA, see S3 Table) for the dataset of the 9,244 unique localities (Fig 1), and the jackknife test of variable importance calculated (for each species) by MaxEnt (see S1 Table)–used frequently to identify those variables with important individual effects (see Wu [68], Berger [69] and Elith [43] for a complete description of this statistical method). We extracted the values for these two variables from 10,000 pixels randomly selected from the modeled TDFs prediction (i.e. predicted present pixels), and we compared the relationship between these two variables considering: (a) each of the locality records and (b) the potential ecological TDFs’ distributions according to the sums of models per pixel.

### Analysis of the ecological and geographical patterns of TDFs

The geographical patterns of species predictions based on their distributional range and habitats or life forms were compared using the three approaches described above, and we characterized their ecological patterns according to major taxonomic groups. These characterizations were used to compare patterns of assemblage variation as projected by the three modeling strategies used (birds, plants and all species combined). Due to differences in ecological and geographical patterns obtained among the three strategies, we established the "all species combined" as the best modeling strategy to use in the reconstruction of TDFs for subsequent analyses (see Results).

To test how much niche overlap (i.e. similarity) among species should occur in order to define the distributions of the Mexican TDFs and to evaluate the pre-established distribution limits among vegetation types, we analyzed the percentage prediction of the primary TDFs map, as well as the geographical overlap with the surrounding ecosystems. For this analysis we followed the classification of Mexican terrestrial ecosystems by Challenger and Soberón [33] that include the natural vegetation range map by INEGI [24]: Temperate Forest (TF), Xerophyte Scrub Forests (XSF), Wetland Forests (WF), Grassland (Gr), Cloud Forests (CF), and Mangrove Swamp (MS). First, we summed the 30 ENMs of species at same time to obtain the consensus map (i.e. modeled TDFs), and then we analyzed the properties of prediction based on the relation to each possible number of species combinations (i.e. the numbers of species per pixel [from 1 to 30 species] independently of the species’ identity), following three approaches: (1) calculating the percentage of the primary TDFs map predicted by the modeled TDFs (% Prediction = [modeled TDFs / primary TDFs map] x 100); (2) establishing the proportion of modeled area with at least “*n*” models (independently of species combinations) that corresponds with the primary TDFs map and the surrounding vegetation types or ecosystems, by calculating the overlap (in pixels) of the predicted area that coincided with each ecosystem; and (3) calculating the proportion of pixels of determined combinations of summed models (from 1 to 30) that correspond with each ecosystem (overlap in pixels). Finally, based on the values obtained from these three analyses, we used an ANOVA test (R software; http://www.r-project.org/) between two groups of combinations (< 10 and ≥ 10; See Results) to evaluate the minimum number of species models required to rebuild the TDFs and to represent their heterogeneous climatic matrix of conditions.

Additionally, the differences between the selected final modeled TDFs (considering the diverse chosen thresholds), the single-ecosystem model map, and the primary TDFs map can be better explained by the existence of transition zones among the ecosystems (and not considered by the last two maps). Thus, we explored the shifts in ecological patterns with other closely-related ecosystems in ecological terms: TF and XSF (see Results). For these analyses we extracted the values for the 19 climatic variables from 1,000 random representative points of each ecosystem type and conducted a PCA and a Discriminant Analysis (DA) using the SPSS ver.19 [70]. These values were also compared with the 100 independently verified TDF occurrence localities.

## Results

### ENMs of species and single-ecosystem model

All ENMs generated for the selected species showed high values for the AUC (0.926–0.990) and AUC-ratios (1.23–1.93; *p* <0.01), with a mean rate of testing omission at 4.65% for species (S1 Table), which indicated that models of the species’ climatic niches were adequate. On the other hand, the single-ecosystem model obtained based on randomly chosen training points within the TDFs polygon showed AUC value of 0.924 and an AUC-ratio of 1.71 ± 0.03 (*p* <0.001), with a testing omission value of 4.75%.

### TDFs reconstruction based on autoecological niche approach

#### 1. Comparing the predicted ecological distribution of each species

**Fig 2:**
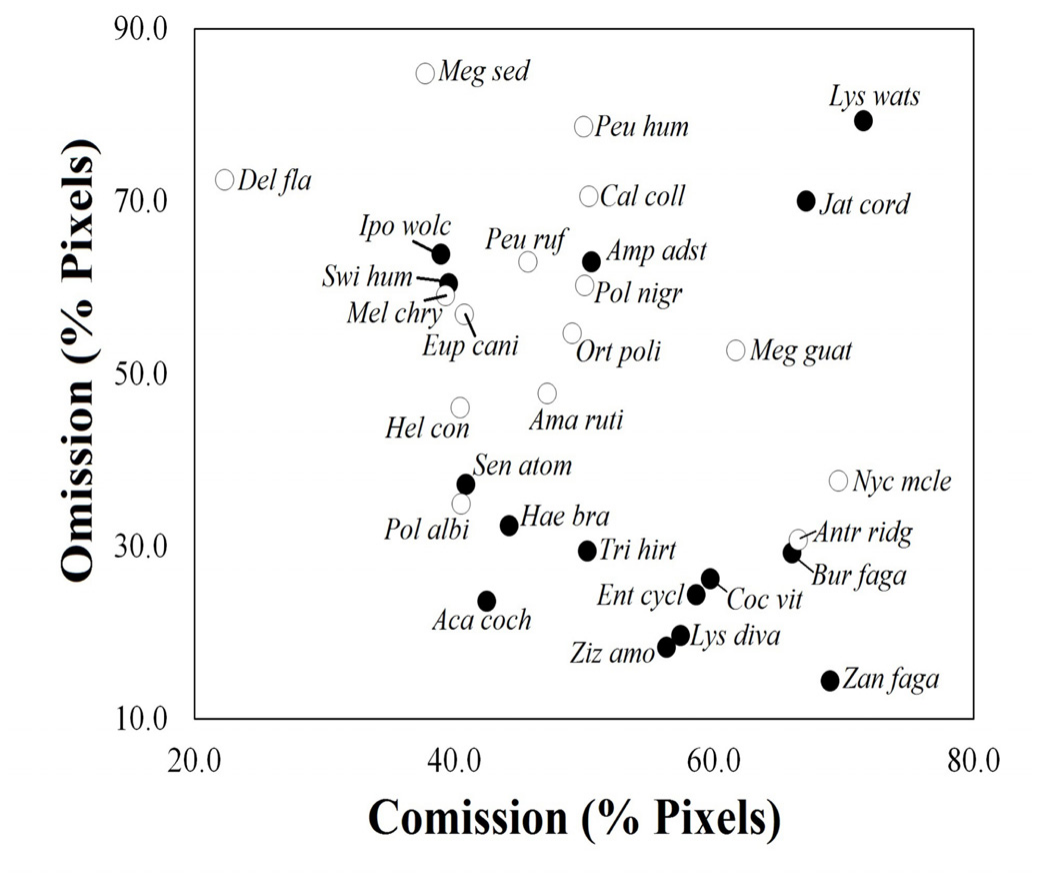
Combined values of omission and commission from the species modeled considering the potential distribution maps of Mexican TDFs. Values were presented in percentage based on primary vegetation maps of Mexico (INEGI, 2003). Name abbreviations are used to refer to each species (See Table 1).

In the estimation of the omission and commission errors for predicting of primary TDFs map based in the individual species' models, we detected an inverse relationship between birds and plants, with plants showing lower omission and higher commission values and birds showing the opposite pattern (Fig 2). For instance, birds in general appeared to be the most restricted to the TDFs (with the exception of *Antrostomus ridgwayi* and *Nyctiphrynus mcleodii*). In contrast, six species of plants (*Bursera fagaroides*, *Cochlospermum vitifolium*, *Enterolobium cyclocarpum*, *Lysiloma divaricatum*, *Zanthoxylum fagara* and *Ziziphus amole*) showed higher commission values and lower omission values. We did not observe grouping patterns among predictions for species based on their trophic guilds, life forms, or biogeographical origin. Lastly, the absence of species with both low omission and commission values suggests that species models avoided overfitting (Fig 2).

#### 2. Considering the sums of species models using three strategies of combination

**Fig 3:**
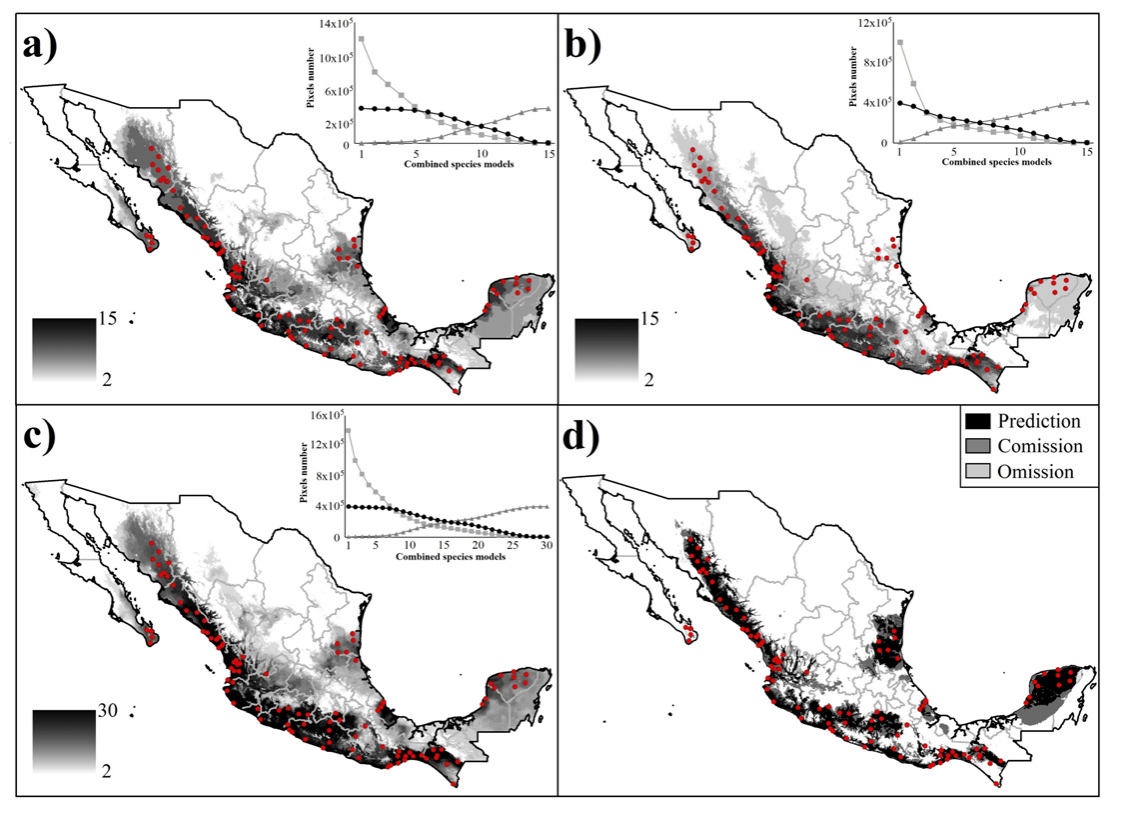
Consensus maps of TDFs in Mexico representing the sum of Ecological Niche Models for the species modeled. Maps from (a) to (c) represent the Mexican TDFs reconstruction by the accumulation of species modeled (from pale to dark black shading indicates accumulation of models). Map (d) represent the TDFs distribution based on a single-ecosystem model approach. White dots represent the known TDFs localities used for evaluation. To the right of maps (a-c), the number of pixels evaluated on the base of TDFs range predicted for each set of species, including the omission (triangles and line dark gray), commission (squares and lines light gray) and prediction (circles and lines black) values for each set. Values were reported based on primary vegetation maps of Mexico (INEGI, 2003). Letters correspond to: sums for only plants species models (a), sums for only bird species models (b), and sums for all species models (c).

The overlap of individual ecological species predictions by combining in each of the three modeling strategies (plants, birds, and all species) resulted in consensus TDFs maps with a gradient of geographic predictions (Fig 3A–3C). These three maps coincided with the primary TDFs map (Fig 1) in identifying four major areas: a continuous strip along the Pacific coast from Sonora to Chiapas (including the Cape region in southern Baja California, and the Bajío region and the Balsas rivers basins in central Mexico), and three discontinuous areas in the Yucatan Peninsula, central Veracruz, and southern Tamaulipas. For TDFs reconstruction based on autoecological niche approach, independently of strategy used in the combination of ENMs, we observed that omission increased when the number of species increased, without any asymptote in the trend line. The opposite pattern was observed for commission errors, which decreased with an increasing number of modeled species (Fig 3A–3C).

#### 3. Comparing the ecological distribution in an two-dimensional space

**Fig 4:**
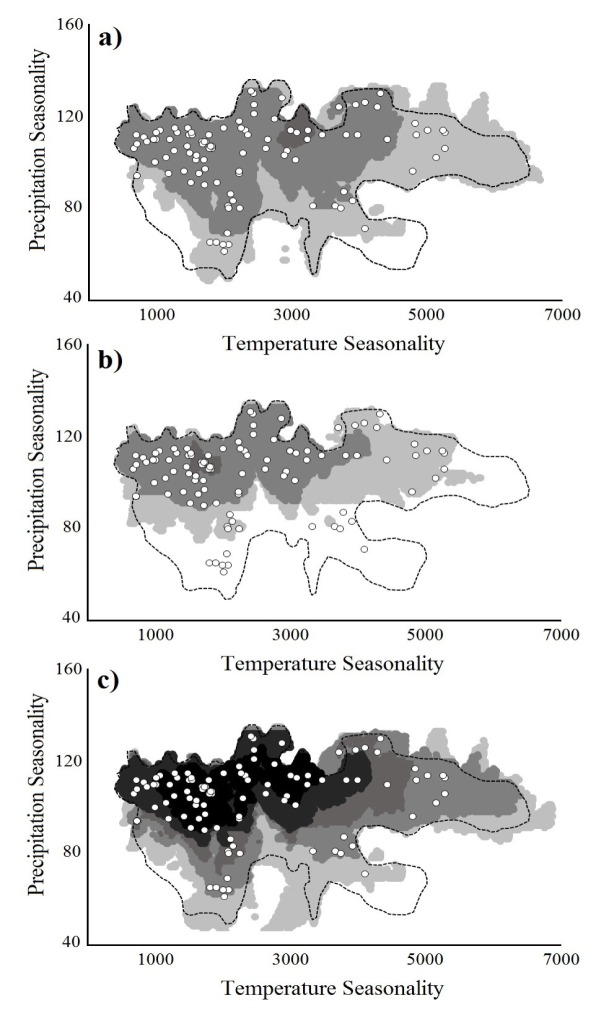
Two-dimensional climatic space (seasonality of precipitation *vs* Temperature seasonality) of the Tropical Dry Forests modeled. In order to simplify the view graphical we presented the results in summed groups of “five by five” species (i.e. 5, 10, 15, 20, 25, 30 species). White dots represent the known TDF localities used for evaluation. Line represents the point’s distribution for single-ecosystem model. (a) Only plants species; (b) Only bird species; and (c) All species.

We observed that ~20 species were mainly influenced by the seasonality in annual temperature and precipitation (S1 Table); which has been defined as the most significant variables to TDFs and/or were used in previous studies [16,39]. Thus, we selected these two variables to represent the ecological space of the modeled TDFs on a two-dimensional scale, comparing them with the independent localities (Fig 4). The comparison of TDFs prediction (in the three strategies) with the 100 independently verified localities showed differences in the number of localities predicted (omission errors) among strategies. In this comparison, we observed that plants possessed a higher range of variation for the values of precipitation and temperature, predicting 100% of the independently verified localities through the overlap of five species models, 81% with 10 models, and 4% with all species models (Fig 4A). In contrast, birds showed preference for areas with lower variation in the precipitation values, with the overlap of five species models predicting 86% of TDFs localities, and the overlap of 10 and 15 species models predicting 68% and 16%, respectively (Fig 4B). Finally, the "all species combined" strategy showed that 100% of localities were predicted with the overlap of five species models, 98% with 10 models, 78% with 15 models, 69% with 20 models and <50% when we using 25 or more species’ ENMs (Fig 4C).

**Fig 5:**
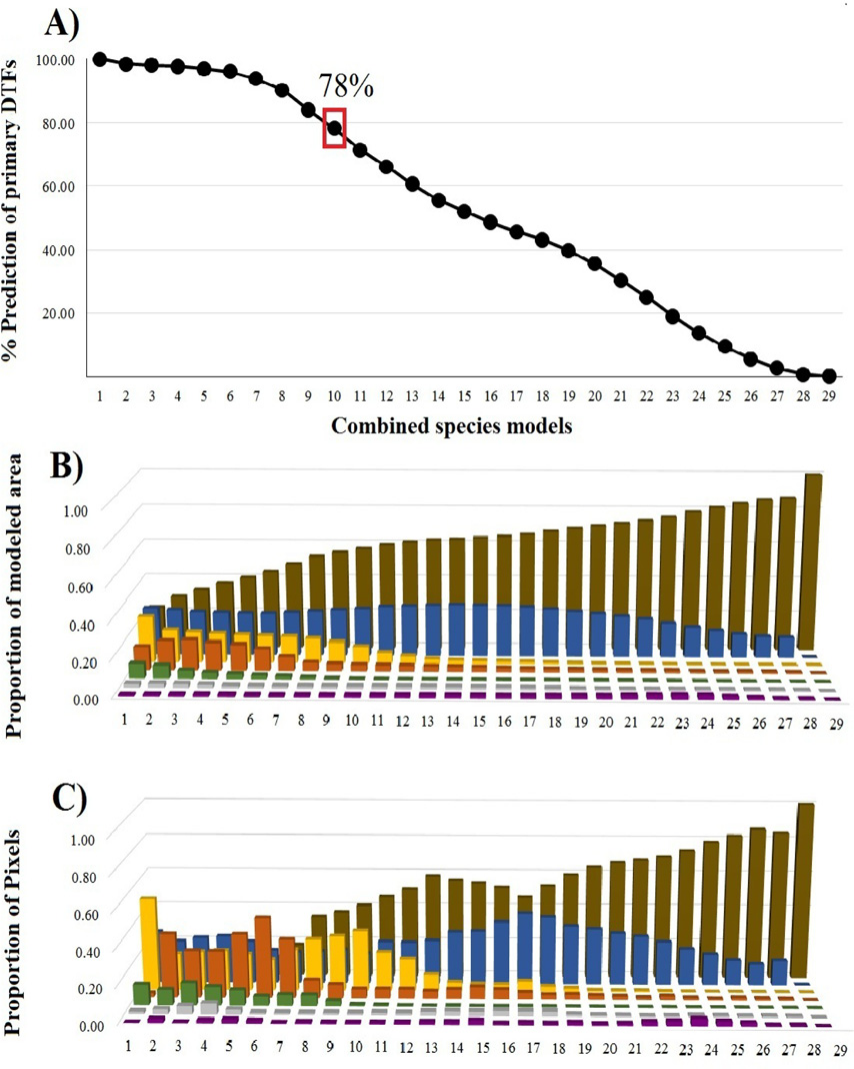
Percentage of prediction and overlap of modeled TDFs in comparison with Mexican ecosystems. (a) Percentages of prediction of primary TDFs maps: note a decreased in the values when the number of species increased, with a prediction of 78% of the TDFs areas by the overlap of 10 species models (independently of species combinations). (b) Proportion of predicted modeled area considered to be TDFs; and (c) Relation between the numbers of species predicted by pixels and each ecosystem type. For both cases (b-c), we observed that proportion of the modeled areas that corresponds with TDFs tends to increase with an increase in the number of combined species models, showing that the combinations of one to nine models are stronger associations with other ecosystems than TDFs. Colors correspond to: TDFs (brown), Temperate Forests (blue), Xerophyte Scrub Forests (yellow), Wetland Forests (orange), Grassland (green), Cloud Forests (grey), and Mangrove Swamp (purple).

Based on the better performance of the "all species combined" strategy during the evaluation process, we observed that the percentage of prediction in relationship to the primary TDFs map tended to decrease as the number of models predicted for species increased (Fig 5A). Predicted values showed that, according to the primary TDFs map, ~97% of areas were predicted with the overlap of five species models, ~78% with 10 models, ~52% with 15 models, and <36% when we overlapped 20 or more species models. Based on comparison with surrounding ecosystems, we observed that the proportion of the modeled areas obtained that corresponded to TDFs increases with an increase in the number of combined species models (Fig 5B), resulting in a smooth transition in the slope displaying the overlap of 10 species. Regarding the relationship between the proportions of pixels and the number of combined models and ecosystems (Fig 5C): from one to nine models we observed higher proportions of associations with other ecosystems than to the TDFs. In addition, when we compared the two groups (< 10 and ≥ 10 species) the ANOVA test showed a significant difference between the proportion of modeled area (F = 71.74, p < 0.001) and the proportions of pixels based on combined models (F = 119.7, p < 0.001), suggesting that although smaller areas are predicted with an increase in the combination of models, these areas are more ecologically similar to what it is considered to be TDFs (as defined in Material and Methods section). Finally, our estimation of the TDFs potential areas based on the threshold of combined models (≥10 species) was of ~561,000 pixels.

### TDFs reconstruction based on single-ecosystem model

The climatic model obtained based on randomly chosen points in the TDFs polygon showed an extent of occurrence of ~588,000 pixels (considering a value of 0.275 [MaxEnt logistic value] as FOV5 threshold; Fig 3D). This model showed a prediction of ~367,000 (62.37%) pixels in the primary TDFs map, with a high (~221,000 pixels) commission value. Additionally, this model predicted the 90% of independently verified localities in the two-dimensional scale (Fig 4).

### Analysis of the ecological-climatic patterns and boundaries of TDFs

The differences among the three maps (modeled TDFs, single-ecosystems model, and primary TDFs) were observed particularly along the northern limits of the Cape (Baja California) and the Sonora-Sinaloa regions, as well as for the Bajío and the southern Pacific Coast regions, which may indicate a potentially larger TDFs extension than the reported by that primary TDFs map. We observed that commission values in models obtained (for both approaches) overlaps with other primary ecosystems, mainly TF and XSF (Fig 5), which suggests a similar climatic relationship among them.

**Fig 6:**
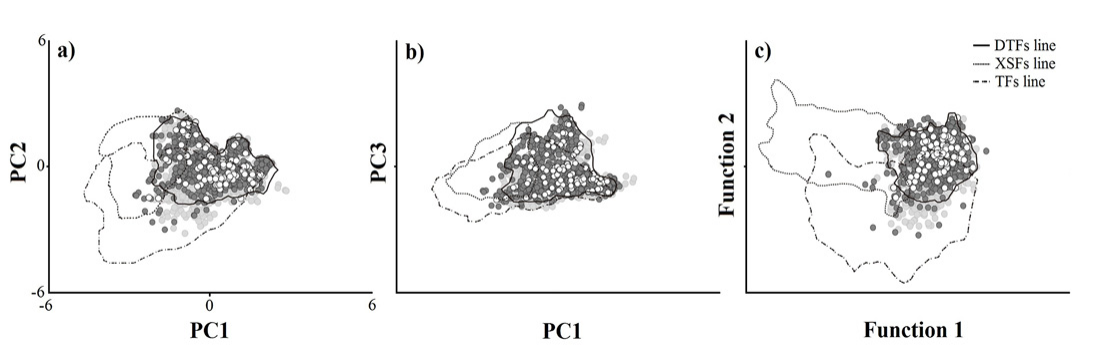
Climate description in the potential distribution of the Mexican TDFs. Points represents the modeled TDFs (light gray), the primary TDFs (dark-gray) and the known TDF localities (white). Letters correspond to: Principal Components Analysis (a-b) and Discriminant Analysis (c). Lines represent the point’s distribution for single-ecosystem model (TDFs line), the Temperate Forests (TFs line) and Xerophyte Scrub Forests (XSFs line).

Finally, the PCA showed that the first three principal components explained 75.78% of the variance observed among the ecosystems, while the DA showed two functions that accounted for 97.9% of the variation and a correct classification of ~49.00% of the cases (S4 Table). However, despite the unique climatic environment corresponding to each ecosystem, there is an important overlap between the selected TDFs points (including those for the single-ecosystem model, the primary TDFs map, and the independently verified localities) and the two comparison ecosystems (Fig 6). This pattern allowed us to consider the existence of transition zones among these ecosystems with similar ranges of temperature and precipitation values.

## Discussion

### TDFs reconstruction: Autoecological niche approach *vs*. Single-ecosystem model

Applications of ENMs at the ecosystem level for conservation purposes are frequently based on mapping the location of climatically suitable areas or investigating the historical distribution by modeling entire vegetation communities or biomes [16,17]. However, attempts to estimate the distribution of entire ecosystems based on single-ecosystem model produce misleading results in the size of ecosystems and their ecological and geographical distribution [12,13,21], due to the potential mismatch between individual species distributions and ecosystem boundaries [13,71], as well as due to the fact that these methods do not consider individual requirements and responses of species to local climatic conditions [3,13,15].

In this study, based on the two verifications (primary vegetation and independent localities), we successfully modeled the distribution of Mexican TDFs using a number of bioclimatic variables and a set of 30 co-distributed species of plants and birds. Despite that number of species is low respect to the high diversity reported for this ecosystem (~6,000 species of plants and 300 species of birds for Mexico [33,36,41]), our study supports the idea that is not necessary to analyze all distributions of occurring species. Our approach includes the assumptions that, to some extent, biotic interactions and abiotic factors have an effect on species and populations at micro and macro ecological scales, respectively; and that species share similar ecological requirements, particularly those that are ecologically restricted [3,5,12,15,21]. Thus, the distribution of particular communities or ecosystems can be reconstructed at broad geographical scales using environmental variables from a low number of species [3,13,15], providing a practical alternative when time and resources are limited.

Considering communities and ecosystems as assemblages of species that could dissociate and associate according to climatic conditions in a spatial-temporal context [8,10,21,72], the autoecological approach represents an appropriate method (compared to single-ecosystem model) to study the historical–past, present and future–distribution of these biological associations, because it considers the individual response of species to local climate conditions [15,18,72], assuming–to some extent–that communities and ecosystems are not static in time and space [8]. However, is important to note that albeit climate is the key limiting factor in the distribution of species and communities at macro ecological scales, the differences obtained between major taxonomic groups supported the idea that some of them might be more geographically and/or ecologically restricted than others [21,23,73,74]. This suggests that throughout the TDFs distribution we can find a gradient of species richness with similar ecological requirements that are linked by a geographic mosaic of evolutionary convergences [12,21,26,28].

#### 1. Ecological and geographical patterns of species and TDFs’ ecosystem

Despite the fact that most of the selected species generally appeared to coincide geographically and ecologically, the species’ individual models presented different degrees of omission and commission errors when compared with the distribution of the primary TDFs map (with differences among the taxonomic groups; Figs 2 and 4). This resulted in a loss of information on the dimensions and distributions of these forests, which confirmed that species may be limited by different environmental parameters throughout their distributional range. Each species possessed a particular shape and optimum range along a given environmental gradient, according to physiological and morphological traits specific to each species [23,74,75], as well as by biogeographic factors that determine their ecological requirements [5,15,18].

From an ecological perspective, our study showed that seasonality of precipitation was the most important variable that restricted species distribution, particularly in birds (Fig 4). The selected plants possessed wider ecological ranges than birds, which suggests that plants shared a similarly large portion of their ecological niche, perhaps because they share mechanisms allowing them to rapidly adapt to variable climatic conditions [76,77], where birds might use different strategies based on their dispersal capabilities [78]. TDFs present a heterogeneous and widely distributed vegetation type, and their different variants show particular physiological and phenological adaptations (such as senescence), allowing them to be resilient to seasonal and multi-year deficits in soil moisture, which in turn also influences the expansion of the distributional limits of species that ultimately also contributes to the determination of their composition in specific regions [79,80]. In contrast, the restricted ecological space observed in birds could be explained by the dependency of animals on water, forcing them to change or to adjust their activity patterns, the use of food resources, and in some cases, the seasonal movement to different ecosystems [78,81,82]. We interpreted this last condition as a measure of nestedness for climatic requirements, where birds’ requirements are intertwined to those of plants that co-occurring in the TDFs [72,83,84]. Thus, based on this predictive behavior and the commission and omission values among the strategies that were applied, we noted that the inclusion of more taxa generate a decreasing of overprediction and an increasing of areas with the most typical TDFs climatic conditions.

Furthermore, if the geographical associations among species' niches are different throughout the distribution of TDFs, then the inclusion of more species models would allow us to find more appropriately–considering for instance conservation goals–the minimum set of species (based on a major number of combinations) that would better represent the TDFs’ distribution both ecologically and geographically. However, it is important to consider that we only included two taxonomic groups and few contrasting life forms or trophic guilds; thus, the use of other species with different categories (e.g. herbaceous, vines or rapacious), as well as species with specific ecological interactions (e.g. mutualism, parasitism) should be also explored as an alternative to improve the performance of the ENM reconstructions.

### Ecosystems’ boundaries based on autoecological niche of species

An important controversy exists on the perception of communities: *are they an organized system of recurrent species or a random set of species with minimal integration*? [8,10]. From a simpler view, if we suppose that communities are natural units, the contact between two different associations must be clear and discontinuous, and the species that conform each community must show similar geographic distributions and boundaries. It is noteworthy in our study that when areas showed climatic values that varied from the most typical climatic conditions associated with the TDFs (see Trejo [39]), the delimitation of this ecosystem in relation to the surrounding ecosystems became a challenge. For example, if conditions tended to be drier or wetter, the shift to neighboring ecosystems such as drier forests (i.e. XSF and TF; see Rzedowski [36,37], and Trejo [38,39]; Figs 5 and 6), greatly hindered the delimitation of the distribution of TDFs. For instance, these particular conditions were observed clearly for the Baja California TDFs (Cape forests), Sonora (Sonora Desert), the Balsas rivers basins areas, and the northern Gulf of Mexico (Tamaulipas-Veracruz forests). Thus, framed from an ecological perspective, the application of the ENM approach for the reconstruction of ecosystems reveals, particularly toward the borders, a gradual geographic and ecological interchange among species of surroundings biological associations.

The overlap in ecological space observed among the three compared ecosystems indicated that there are transitional areas among the TDFs with its neighboring XSF and TF, and these areas might represent the individual responses of species to similar ranges of temperature and precipitation values along the transitional areas of such ecosystems. Thus, it is possible to find areas where deciduous, semi-deciduous, and evergreen forests co-occur based on their shared species [33,39,40,80]. These results support the idea of communities as associations that are not separated but rather continuous gradations, particularly towards the borders, where species groups are not constant from one place to another [3,8,10]. Although two ecosystems may share species, their composition may differ greatly, and these differences in accumulation patterns might be explained by diverse evolutionary histories throughout different geographic regions [85] and according to the geographic mosaic of species convergence due to climatic conditions [12,21,73]. In this case, the reconstruction of communities and ecosystems at macro-geographic or ecological scales must be developed using closely related or ecologically similar species [5,20,73].

### Final considerations

This study suggests a re-evaluation and re-interpretation of the current ecological and geographical distribution of Mexican TDFs. In spite of established preferences for particular environmental conditions, we found that diverse areas are not easily distinguishable and have partial overlap with other ecosystems; suggesting that the new approach offers an alternative for the reconstruction of the environmental conditions suitable for TDFs development and other ecosystems. However, dispersal and history, together with spatial competition with other assemblages may prevent that these predictions comes true in some areas. Thus, we propose a rethinking of the definition of ecosystem limits that would promote a better understanding of the real nature of gradual transition areas among ecosystems, instead of artificial limits that only satisfy the need for classification, rather than the description of the fundamental structure of communities. This will allow us to understand the dynamic association of species in order to reconstruct, based on climate, their common distributional patterns, and to measure the potential effect of future scenarios of climate change on the functional processes of ecosystems [2,3,20].

Finally, although a thorough discussion of the use of the autoecological approach to improve biodiversity conservation is beyond the scope of this study, we contend that use of ENM provides testable predictions for the reconstruction of ecosystems and will contribute significantly to more efficient strategies for conservation, management, and restoration [1,3,5,15]. The main lesson emerging from these results is that spatial patterns influence the distribution of biodiversity at a collective level, and this may vary significantly in geographical space. Our results clearly suggest that assemblages of TDFs species (and their spatial variation) should also be assessed, especially considering biogeographical studies of species associations throughout regions and the future impacts of climate change on the distribution and biodiversity of ecosystems [3,20,21,74,86]. Thus, this perspective should be used to incorporate the variability in the strength of assembly drivers over geographical and environmental spaces in ecosystems and their transitional areas.

## Supporting Information

S1 Table Percentage of contribution of each environmental variable used and validation values obtained for the species models generated. (DOCX)

S2 Table Locality records of Tropical Dry Forests in Mexico used to evaluate ENMs of species for the prediction of TDFs. (DOCX)

S3 Table Character loading and percentage of explained variance for Principal Components I–III for the dataset of the 9,244 unique localities. (DOCX)

S4 Table Character loading and percentage of explained variance for Principal Components I–III and Discriminant Analysis for environmental variables. (DOCX)
