# Supplementary material for: Reconstructing the Mexican Tropical Dry Forests via an Autoecological Niche Approach: Reconsidering the Ecosystem Boundaries
Source: PLoS One. 2016 Mar 11;11(3):e0150932. doi: 10.1371/journal.pone.0150932 (PMC4788342; doi:10.1371/journal.pone.0150932)
Supplement: S1 Table — (DOCX) [file pone.0150932.s001.docx]

**S1 Table. Percentage of contribution of each environmental variable used and validation values obtained for the species models generated**. Bold numbers indicate highest percentage of contribution (the two most important variables).

| **Species** | **PERCENTAGE OF CONTRIBUTION** | | | | | | | | | | | | | | | | | | | **VALIDATION** | | |
| --- | --- | --- | --- | --- | --- | --- | --- | --- | --- | --- | --- | --- | --- | --- | --- | --- | --- | --- | --- | --- | --- | --- |
|  | BIO1 | BIO2 | BIO3 | BIO4 | BIO5 | BIO6 | BIO7 | BIO8 | BIO9 | BIO10 | BIO11 | BIO12 | BIO13 | BIO14 | BIO15 | BIO16 | BIO17 | BIO18 | BIO19 | AUC | AUC ratio | Z score* |
| **Birds** |  |  |  |  |  |  |  |  |  |  |  |  |  |  |  |  |  |  |  |  |  |  |
| *Amazilia rutila* | 3.0 | 0.8 | 5.1 | 4.1 | 0.8 | 2.1 | **10.6** | 3.3 | 0.4 | 0.1 | **47.8** | 1.3 | 0.0 | 1.3 | 8.5 | 0.3 | 6.0 | 2.5 | 2.0 | 0.945 | 5.00 | 1.56 |
| *Calocitta colliei* | 0.2 | 0.2 | 3.6 | 7.4 | 2.3 | 4.3 | 0.1 | 0.3 | 3.7 | 0.0 | 0.4 | 0.3 | 0.2 | 0.9 | **51.8** | 0.3 | 0.2 | **23.0** | 0.8 | 0.971 | 5.00 | 1.75 |
| *Antrostomus ridgwayi* | 0.0 | 0.2 | 0.1 | 2.0 | 2.3 | 7.9 | 1.1 | 0.0 | 2.8 | 3.0 | 0.0 | 5.9 | 11.0 | **15.9** | **42.5** | 1.3 | 0.9 | 1.3 | 1.8 | 0.946 | 5.00 | 1.50 |
| *Deltarhynchus flammulatus* | 0.5 | 2.9 | 0.1 | 3.8 | 0.0 | 8.1 | 0.0 | 0.9 | 1.9 | 0.3 | **43.2** | 0.0 | 0.1 | 11.4 | **21.3** | 2.6 | 1.8 | 0.7 | 0.3 | 0.988 | 3.40 | 1.85 |
| *Eupsittula canicularis* | 0.0 | 0.8 | 0.5 | 0.6 | 0.4 | 8.0 | 7.2 | 0.1 | 2.2 | 1.1 | **34.7** | 2.0 | 0.3 | 0.5 | **38.3** | 1.6 | 0.2 | 1.2 | 0.2 | 0.953 | 4.70 | 1.54 |
| *Heliomaster constantii* | 0.1 | 1.0 | 6.4 | 1.6 | 0.4 | 13.1 | 1.5 | 0.5 | 2.5 | 0.3 | **17.8** | 1.6 | 0.7 | 2.0 | **38.6** | 8.8 | 0.3 | 1.9 | 0.8 | 0.959 | 4.50 | 1.70 |
| *Megascops guatemalae* | 0.1 | 4.0 | 2.4 | 4.8 | 1.0 | 0.2 | **41.5** | 1.4 | 0.8 | 0.3 | **19.0** | 1.0 | 0.5 | 0.9 | 1.7 | 2.1 | 0.0 | 17.1 | 1.5 | 0.962 | 4.20 | 1.46 |
| *M. seductus* | 0.0 | 0.4 | 0.0 | **45.1** | 0.5 | 3.8 | 3.0 | 0.0 | 0.0 | 0.0 | 1.4 | 0.0 | 2.1 | **13.9** | 0.5 | 9.0 | 12.1 | 4.0 | 4.5 | 0.990 | 4.00 | 1.79 |
| *Melanerpes chrysogenys* | 1.0 | 0.1 | 2.6 | 10.9 | 0.2 | 4.6 | 7.6 | 0.7 | 4.3 | 0.2 | **14.1** | 1.7 | 0.3 | 0.2 | **48.1** | 0.4 | 0.1 | 2.6 | 0.1 | 0.955 | 4.90 | 1.72 |
| *Nyctiphrynus mcleodii* | 0.0 | 0.9 | 2.2 | 0.0 | 0.2 | 1.6 | 2.7 | 0.0 | 0.1 | 7.3 | 0.0 | 0.0 | 0.0 | **22.5** | 17.0 | **43.9** | 0.0 | 1.5 | 0.1 | 0.961 | 5.00 | 1.71 |
| *Ortalis poliocephala* | 5.3 | 0.3 | 1.1 | **24.7** | 1.7 | 2.2 | 12.5 | 0.8 | 1.8 | 1.8 | 1.2 | 2.8 | 0.5 | 0.1 | **41.7** | 0.0 | 0.0 | 1.5 | 0.0 | 0.963 | 4.50 | 1.59 |
| *Peucaea humeralis* | 0.1 | 1.1 | 0.2 | **39.0** | 0.1 | 6.5 | 5.5 | 0.5 | 0.3 | 0.0 | 0.3 | 0.0 | 3.4 | 0.2 | **30.3** | 2.7 | 6.9 | 2.6 | 0.5 | 0.983 | 4.40 | 1.83 |
| *P. ruficauda* | 0.0 | 0.7 | 2.8 | **38.2** | 1.2 | 0.6 | 0.1 | 0.2 | 1.4 | 2.8 | 1.8 | 2.3 | 0.1 | 0.1 | **43.8** | 0.2 | 0.5 | 0.1 | 3.0 | 0.957 | 4.80 | 1.77 |
| *Polioptila albiloris* | 2.6 | 1.5 | 7.3 | 8.7 | 0.4 | 12.7 | 7.7 | 0.9 | 0.4 | 0.6 | **23.0** | 1.9 | 2.1 | 0.8 | **12.9** | 3.3 | 5.9 | 1.4 | 5.9 | 0.955 | 4.80 | 1.45 |
| *P. nigriceps* | 2.8 | 1.7 | 2.2 | 3.1 | 0.3 | 0.2 | 1.1 | 3.7 | 4.2 | 0.2 | 2.7 | 0.4 | 0.9 | 0.1 | **58.6** | 0.1 | 0.4 | **14.7** | 2.5 | 0.978 | 4.40 | 1.55 |
| **Plants** |  |  |  |  |  |  |  |  |  |  |  |  |  |  |  |  |  |  |  |  |  |  |
| *Acacia cochliacantha* | 0.0 | 0.2 | 0.1 | 2.9 | 2.2 | 3.3 | 1.7 | 0.6 | **14.6** | 1.5 | 20.4 | 7.9 | 0.9 | 0.7 | **37.7** | 0.7 | 0.1 | 3.1 | 1.5 | 0.954 | 4.4 | 1.42 |
| *Amphipterygium adstringens* | 1.6 | 0.1 | 0.0 | **40.2** | 0.5 | 1.1 | 0.1 | 0.2 | 0.3 | 0.0 | 9.8 | 6.1 | 0.0 | 0.2 | 11.7 | 0.0 | 7.7 | 0.5 | **20.0** | 0.974 | 3.6 | 1.51 |
| *Bursera fagaroides* | 0.4 | 1.1 | 10.1 | 10.2 | 0.7 | 1.8 | 0.2 | 0.2 | 0.3 | 0.2 | **16.7** | 4.5 | 2.2 | 0.6 | **41.9** | 0.1 | 0.2 | 2.7 | 5.9 | 0.929 | 4.9 | 1.23 |
| *Cochlospermum vitifolium* | 1.4 | 0.8 | 0.5 | **25.5** | 0.0 | **32.5** | 2.4 | 2.5 | 2.1 | 0.0 | 3.7 | 0.0 | 0.5 | 0.7 | 2.4 | 22.9 | 0.3 | 0.1 | 1.5 | 0.936 | 5.4 | 1.55 |
| *Enterolobium cyclocarpum* | 1.6 | 0.1 | 1.4 | **24.7** | 0.0 | **38.9** | 0.0 | 0.0 | 1.1 | 0.0 | 2.2 | 5.1 | 0.2 | 2.1 | 6.9 | 11.9 | 0.0 | 0.1 | 3.8 | 0.946 | 4.9 | 1.47 |
| *Haematoxylum brasiletto* | **17.0** | 0.0 | 0.2 | 6.5 | 4.5 | 7.5 | 0.0 | 0.4 | 2.7 | 1.9 | 0.6 | 0.0 | 1.8 | 0.6 | **53.6** | 0.7 | 0.7 | 0.3 | 0.9 | 0.926 | 4.7 | 1.52 |
| *Ipomoea wolcottiana* | 0.0 | 0.1 | 0.0 | **31.5** | 1.1 | 10.2 | **24.2** | 1.4 | 0.7 | 0.0 | 0.1 | 0.4 | 0.0 | 1.8 | 10.4 | 1.1 | 0.0 | 0.2 | 16.7 | 0.986 | 6.0 | 1.75 |
| *Jatropha cordata* | 0.0 | 0.4 | 0.0 | **9.7** | 1.9 | 5.5 | 3.5 | 2.3 | 6.3 | 0.0 | 0.0 | 1.4 | 6.4 | 3.2 | **55.2** | 0.2 | 0.3 | 1.7 | 1.9 | 0.975 | 5.0 | 1.69 |
| *Lysiloma divaricatum* | 2.1 | 0.8 | 1.9 | 3.0 | 2.4 | 9.0 | 0.6 | 0.4 | 0.8 | 0.8 | **26.9** | 3.8 | 5.0 | 0.5 | **39.1** | 1.0 | 0.1 | 0.8 | 1.0 | 0.945 | 4.3 | 1.34 |
| *Lysiloma watsonii* | 0.0 | 0.6 | 1.8 | **22.6** | 0.0 | 0.0 | 0.0 | 10.3 | 6.3 | 0.1 | 0.1 | 0.1 | 0.0 | 4.5 | **35.3** | 0.4 | 0.2 | 0.0 | 17.9 | 0.987 | 5.0 | 1.93 |
| *Senna atomaria* | 1.7 | 0.7 | 1.3 | **17.3** | 0.2 | **23.5** | 5.6 | 1.2 | 3.8 | 1.5 | 7.3 | **17.3** | 4.7 | 0.3 | 1.7 | 2.4 | 2.8 | 3.0 | 3.7 | 0.955 | 4.6 | 1.52 |
| *Swietenia humilis* | 0.7 | 0.8 | 1.7 | **35.7** | 0.0 | 6.1 | 0.1 | 0.0 | 0.2 | 0.0 | 7.8 | 0.1 | 0.0 | **16.6** | 5.3 | 15.0 | 2.6 | 0.7 | 6.7 | 0.967 | 4.0 | 1.81 |
| *Trichilia hirta* | 3.3 | 0.3 | 0.2 | 19.0 | 0.1 | **23.4** | 3.1 | 1.0 | 0.3 | 1.7 | 12.1 | 0.4 | 0.0 | 0.7 | 3.9 | **27.6** | 0.3 | 0.4 | 2.3 | 0.944 | 4.5 | 1.42 |
| *Zanthoxylum fagara* | 1.5 | 0.1 | 3.1 | **18.2** | 0.0 | **34.1** | 0.8 | 0.0 | 0.3 | 2.4 | 0.0 | 5.1 | 12.0 | 0.5 | 17.6 | 0.1 | 0.1 | 2.5 | 1.5 | 0.933 | 4.8 | 1.62 |
| *Ziziphus amole* | 1.2 | 2.5 | 2.0 | 2.2 | 0.2 | 13.6 | 1.3 | 1.0 | 1.5 | 0.0 | 13.6 | 3.6 | 0.0 | 3.3 | **31.8** | 3.8 | 0.9 | 0.1 | **17.5** | 0.962 | 5.0 | 1.56 |
| **Means** | 1.6 | 0.8 | 2.0 | **15.4** | 0.9 | 9.5 | 4.9 | 1.2 | 2.3 | 0.9 | 11.0 | 2.6 | 1.9 | 3.6 | **27.0** | 5.5 | 1.7 | 3.1 | 4.2 | 0.960 | 4.65 | 1.60 |

***Values with a significance level of <0.001**
