# Supplementary material for: Reconstructing the Mexican Tropical Dry Forests via an Autoecological Niche Approach: Reconsidering the Ecosystem Boundaries
Source: PLoS One. 2016 Mar 11;11(3):e0150932. doi: 10.1371/journal.pone.0150932 (PMC4788342; doi:10.1371/journal.pone.0150932)
Supplement: S2 Table — (DOCX) [file pone.0150932.s002.docx]

**S2 Table. Locality records of Tropical Dry Forests in Mexico used to evaluate ENMs of species for the prediction of TDFs.** Geographic coordinates are provided in decimal degrees, based on the WGS 84 datum. Source: **Museums**= Collection records from the Global Biodiversity Information Facility database and Atlas of the Birds of Mexico; **Fieldwork**= Localities records obtained from fieldwork; **Literature**= Trejo and Dirzo (2000).

| **ID** | **State / Municipality** | **Latitude** | **Longitude** | **SOURCE** |
| --- | --- | --- | --- | --- |
| 1 | Baja California Sur, La Paz | -110.09013 | 23.84647 | Museums |
| 2 | Baja California Sur, La Paz | -109.83243 | 23.77866 | Museums |
| 3 | Baja California Sur, Los Cabos | -109.90025 | 23.07338 | Museums |
| 4 | Baja California Sur, Los Cabos | -109.80000 | 23.43333 | Museums |
| 5 | Campeche, Calkini | -90.01700 | 20.43300 | Fieldwork |
| 6 | Campeche, Campeche | -90.50500 | 19.78500 | Fieldwork |
| 7 | Campeche, Champotón | -90.70000 | 19.45000 | Fieldwork |
| 8 | Chiapas, Arriaga | -93.94848 | 16.25540 | Museums |
| 9 | Chiapas, Chiapa de Corzo | -93.06131 | 16.78095 | Museums |
| 10 | Chiapas, Jiquipilas | -93.52800 | 16.75800 | Museums |
| 11 | Chiapas, La Concordia | -92.75000 | 15.92000 | Museums |
| 12 | Chiapas, La Trinitaria | -92.13500 | 16.06000 | Museums |
| 13 | Chiapas, Tapachula | -92.40131 | 14.74095 | Museums |
| 14 | Chiapas, Villaflores | -93.36814 | 16.30095 | Museums |
| 15 | Chiapas, Villaflores | -93.32018 | 16.42581 | Museums |
| 16 | Chihuahua, Uruachi | -108.84000 | 27.81200 | Museums |
| 17 | Colima, Colima | -103.69176 | 19.18094 | Museums |
| 18 | Colima, Colima | -103.69157 | 19.18082 | Museums |
| 19 | Guerrero, Acapulco de Juárez | -99.90000 | 16.88300 | Museums |
| 20 | Guerrero, Buenavista de Cuéllar | -99.50700 | 18.41200 | Museums |
| 21 | Guerrero, Chilpacingo de los Bravos | -99.53500 | 17.20200 | Museums |
| 22 | Guerrero, Eduardo Neri | -99.55814 | 17.92095 | Literature |
| 23 | Guerrero, La Unión de Isidoro Montes de Oca | -101.84814 | 17.95095 | Museums |
| 24 | Guerrero, La Unión de Isidoro Montes de Oca | -101.70731 | 17.80830 | Museums |
| 25 | Guerrero, Tecpan de Galeana | -100.93962 | 17.38709 | Museums |
| 26 | Guerrero, Xochistlahuaca | -98.23000 | 16.78000 | Museums |
| 27 | Guerrero, Zirándaro | -101.01100 | 18.28500 | Literature |
| 28 | Jalisco, Cabo Corrientes | -105.45502 | 20.24996 | Museums |
| 29 | Jalisco, La Huerta | -105.07814 | 19.52095 | Museums |
| 30 | Jalisco, Puerto Vallarta | -105.11814 | 20.75095 | Museums |
| 31 | Michoacán de Ocampo, Apatzingan | -102.46774 | 19.09943 | Museums |
| 32 | Michoacán de Ocampo, Aquila | -103.52257 | 18.56499 | Museums |
| 33 | Michoacán de Ocampo, Arteaga | -101.94926 | 18.26026 | Literature |
| 34 | Michoacán de Ocampo, Gabriel Zamora | -101.94400 | 19.21400 | Literature |
| 35 | Michoacán de Ocampo, Tiquicheo de Nicolás Romero | -100.80000 | 18.83300 | Literature |
| 36 | Michoacán de Ocampo, Turicato | -101.32400 | 18.87700 | Literature |
| 37 | Morelos, Jiutepec | -99.17814 | 18.92095 | Literature |
| 38 | Morelos, Tepalcingo | -98.93700 | 18.53200 | Museums |
| 39 | Nayarit, Bahía de Banderas | -105.40814 | 20.89095 | Museums |
| 40 | Nayarit, Del Nayar | -104.85348 | 21.79841 | Fieldwork |
| 41 | Nayarit, Del Nayar | -104.71614 | 21.86929 | Fieldwork |
| 42 | Nayarit, Del Nayar | -104.55000 | 22.25000 | Museums |
| 43 | Nayarit, Rosamorada | -104.93544 | 22.04872 | Fieldwork |
| 44 | Nayarit, Ruiz | -105.06700 | 21.93300 | Fieldwork |
| 45 | Nayarit, San Blas | -105.21862 | 21.58490 | Museums |
| 46 | Nayarit, Tepic | -104.76600 | 21.41100 | Museums |
| 47 | Nayarit, Xalisco | -105.05814 | 21.43095 | Museums |
| 48 | Oaxaca, Asunción Ixtaltepec | -94.89125 | 16.59891 | Museums |
| 49 | Oaxaca, Ixpantepec Nieves | -98.01400 | 17.56000 | Museums |
| 50 | Oaxaca, Magdalena Tequisistlán | -95.60200 | 16.39700 | Museums |
| 51 | Oaxaca, Salina Cruz | -95.19304 | 16.17039 | Museums |
| 52 | Oaxaca, San Blas Atempa | -95.13700 | 16.38400 | Museums |
| 53 | Oaxaca, San Dionisio del Mar | -94.70975 | 16.38848 | Museums |
| 54 | Oaxaca, San Pedro Huamelula | -96.00418 | 15.85345 | Fieldwork |
| 55 | Oaxaca, San Pedro Tapanatepec | -94.21814 | 16.44095 | Museums |
| 56 | Oaxaca, Santa María Huatulco | -96.16800 | 15.75600 | Fieldwork |
| 57 | Oaxaca, Santiago Astata | -95.57814 | 15.97095 | Fieldwork |
| 58 | Oaxaca, Santiago Jamiltepec | -97.84901 | 16.29106 | Museums |
| 59 | Oaxaca, Santiago Lachiguiri | -95.52000 | 16.68000 | Museums |
| 60 | Oaxaca, Tlacolula de Matamoros | -96.45304 | 16.95039 | Museums |
| 61 | Puebla, Caltepec | -97.47900 | 18.16800 | Museums |
| 62 | Sinaloa, Concordia | -106.06814 | 23.28095 | Museums |
| 63 | Sinaloa, Concordia | -106.06700 | 23.28300 | Museums |
| 64 | Sinaloa, Concordia | -105.93689 | 23.40197 | Museums |
| 65 | Sinaloa, Cosalá | -106.76000 | 24.39600 | Museums |
| 66 | Sinaloa, Culiacán | -107.20200 | 24.87000 | Museums |
| 67 | Sinaloa, El Fuerte | -108.57200 | 26.49200 | Museums |
| 68 | Sinaloa, Escuinapa | -105.94700 | 22.78800 | Museums |
| 69 | Sinaloa, Escuinapa | -105.77000 | 22.92000 | Museums |
| 70 | Sinaloa, Mazatlán | -106.37387 | 23.35821 | Museums |
| 71 | Sinaloa, Mocorito | -107.80400 | 24.99600 | Museums |
| 72 | Sinaloa, Rosario | -105.71045 | 23.01908 | Museums |
| 73 | Sinaloa, Salvador Alvarado | -108.14814 | 25.47095 | Museums |
| 74 | Sinaloa, San Ignacio | -106.84400 | 23.76200 | Museums |
| 75 | Sinaloa, San Ignacio | -106.59131 | 23.69095 | Museums |
| 76 | Sonora, Álamos | -109.11131 | 27.22095 | Museums |
| 77 | Sonora, Álamos | -108.91015 | 27.02021 | Museums |
| 78 | Sonora, Cajeme | -109.73749 | 28.01031 | Museums |
| 79 | Sonora, Navojoa | -109.29000 | 27.08000 | Museums |
| 80 | Sonora, Onavas | -109.39842 | 28.47145 | Museums |
| 81 | Sonora, Quiriego | -109.42554 | 27.69836 | Museums |
| 82 | Sonora, Soyopa | -109.81887 | 28.95972 | Museums |
| 83 | Tamaulipas, Aldama | -98.16700 | 23.23300 | Museums |
| 84 | Tamaulipas, Antiguo Morelos | -99.05349 | 22.51236 | Museums |
| 85 | Tamaulipas, El Mante | -98.56679 | 22.53701 | Museums |
| 86 | Tamaulipas, Gomez-Farías | -99.09152 | 23.03126 | Museums |
| 87 | Tamaulipas, Soto la Marina | -98.21660 | 23.62398 | Museums |
| 88 | Veracruz de Ignacio de la Llave, Actopan | -96.46700 | 19.50000 | Museums |
| 89 | Veracruz de Ignacio de la Llave, Actopan | -96.40554 | 19.57796 | Fieldwork |
| 90 | Veracruz de Ignacio de la Llave, Huatusco | -96.74800 | 19.08400 | Museums |
| 91 | Veracruz de Ignacio de la Llave, Puente Nacional | -96.56053 | 19.28382 | Museums |
| 92 | Veracruz de Ignacio de la Llave, Tampico Alto | -98.01700 | 22.05000 | Museums |
| 93 | Yucatán, Dzilam de Bravo | -88.88389 | 21.38148 | Museums |
| 94 | Yucatán, Dzoncauich | -88.89200 | 21.04700 | Museums |
| 95 | Yucatán, Maxcanu | -90.21700 | 20.68300 | Fieldwork |
| 96 | Yucatán, Mérida | -89.64300 | 21.15700 | Fieldwork |
| 97 | Yucatán, Tizimin | -88.16700 | 21.21700 | Museums |
| 98 | Yucatán, Valladolid | -88.20854 | 20.72818 | Museums |
| 99 | Yucatán, Yaxcaba | -88.82800 | 20.49200 | Museums |
| 100 | Zacatecas, Moyahua de Estrada | -103.20014 | 21.24238 | Museums |
