# Supplementary material for: Reconstructing the Mexican Tropical Dry Forests via an Autoecological Niche Approach: Reconsidering the Ecosystem Boundaries
Source: PLoS One. 2016 Mar 11;11(3):e0150932. doi: 10.1371/journal.pone.0150932 (PMC4788342; doi:10.1371/journal.pone.0150932)
Supplement: S3 Table — (DOCX) [file pone.0150932.s003.docx]

**S3 Table. Character loading and percentage of explained variance for Principal Components I–III for the dataset of the 9,244 unique localities.** Bold numbers indicate highest loadings.

| **Variables** | **Principal Components Analysis** | | | |
| --- | --- | --- | --- | --- |
|  | **PC1** | **PC2** | **PC3** | **Communalities** |
| Annual average temperature | **0.773** | 0.582 | 0.230 | 0.990 |
| Average daily range | -0.506 | 0.324 | -0.476 | 0.975 |
| Isothermality | 0.675 | -0.185 | -0.373 | 0.872 |
| Temperature seasonality | **-0.795** | 0.383 | 0.165 | 0.989 |
| Maximum temperature of warmest month | -0.017 | **0.873** | 0.013 | 0.974 |
| Minimum temperature of coldest month | **0.933** | 0.071 | 0.249 | 0.995 |
| Annual temperature range | **-0.784** | 0.413 | -0.200 | 0.997 |
| Average temperature of wettest quarter | 0.156 | **0.831** | 0.325 | 0.956 |
| Average temperature of driest quarter | **0.732** | 0.599 | 0.103 | 0.908 |
| Average temperature of warmest quarter | 0.219 | **0.895** | 0.279 | 0.989 |
| Average temperature of coldest quarter | **0.942** | 0.211 | 0.041 | 0.993 |
| Annual precipitation | 0.606 | -0.411 | 0.054 | 0.949 |
| Precipitation in wettest month | 0.551 | -0.277 | -0.220 | 0.972 |
| Precipitation in driest month | -0.062 | -0.299 | **0.893** | 0.935 |
| Seasonality of precipitation | 0.022 | 0.286 | **-0.815** | 0.911 |
| Precipitation in wettest quarter | 0.550 | -0.304 | -0.188 | 0.980 |
| Precipitation in driest quarter | -0.102 | -0.272 | **0.913** | 0.968 |
| Precipitation in warmest quarter | 0.141 | -0.245 | -0.048 | 0.822 |
| Precipitation in coldest quarter | -0.401 | -0.011 | **0.713** | 0.809 |
| **Eigenvalue** | 6.089 | 4.102 | 3.636 |  |
| **% variance explained** | 32.048 | 21.592 | 19.139 |  |
| **Cumulative % explained** | 32.048 | 53.639 | 72.778 |  |
