# Supplementary material for: Reconstructing the Mexican Tropical Dry Forests via an Autoecological Niche Approach: Reconsidering the Ecosystem Boundaries
Source: PLoS One. 2016 Mar 11;11(3):e0150932. doi: 10.1371/journal.pone.0150932 (PMC4788342; doi:10.1371/journal.pone.0150932)
Supplement: S4 Table — (DOCX) [file pone.0150932.s004.docx]

**S4 Table. Character loading and percentage of explained variance for Principal Components I–III and Discriminant Analysis for environmental variables.** Bold numbers indicate highest loadings.

| **Variables** | **Principal Components Analysis** | | | | **Discriminant Analysis** | |
| --- | --- | --- | --- | --- | --- | --- |
|  | **PC1** | **PC2** | **PC3** | **Communalities** | **F1** | **F2** |
| Annual average temperature | **0.845** | 0.411 | 0.290 | 0.967 | 0.552 | 0.746 |
| Average daily range | -0.238 | 0.669 | -0.345 | 0.624 | -0.258 | -0.111 |
| Isothermality | 0.690 | -0.106 | -0.309 | 0.583 | 0.374 | -0.165 |
| Temperature seasonality | -0.598 | 0.609 | 0.292 | 0.815 | -0.503 | 0.183 |
| Maximum temperature of warmest month | 0.341 | **0.878** | 0.170 | 0.916 | 0.144 | 0.691 |
| Minimum temperature of coldest month | **0.894** | -0.213 | 0.218 | 0.892 | 0.633 | 0.474 |
| Annual temperature range | -0.511 | **0.761** | -0.067 | 0.845 | -0.475 | 0.036 |
| Average temperature of wettest quarter | 0.421 | **0.720** | 0.438 | 0.887 | 0.282 | 0.669 |
| Average temperature of driest quarter | **0.828** | 0.417 | 0.150 | 0.882 | 0.558 | 0.561 |
| Average temperature of warmest quarter | 0.476 | 0.765 | 0.384 | 0.959 | 0.220 | 0.796 |
| Average temperature of coldest quarter | **0.950** | 0.024 | 0.051 | 0.906 | 0.676 | 0.500 |
| Annual precipitation | 0.652 | -0.467 | 0.040 | 0.644 | 0.559 | -0.287 |
| Precipitation in wettest month | 0.624 | -0.228 | -0.295 | 0.529 | 0.598 | -0.295 |
| Precipitation in driest month | -0.014 | -0.338 | **0.878** | 0.886 | 0.069 | -0.102 |
| Seasonality of precipitation | 0.217 | 0.493 | -0.757 | 0.864 | 0.330 | -0.107 |
| Precipitation in wettest quarter | 0.641 | -0.262 | -0.314 | 0.578 | 0.579 | -0.293 |
| Precipitation in driest quarter | -0.055 | -0.315 | **0.904** | 0.919 | 0.068 | -0.132 |
| Precipitation in warmest quarter | 0.148 | -0.126 | 0.027 | 0.038 | 0.421 | -0.236 |
| Precipitation in coldest quarter | -0.236 | 0.007 | **0.779** | 0.663 | 0.092 | -0.222 |
| **Eigenvalue** | 6.126 | 4.485 | 3.787 |  | 1.808 | 0.981 |
| **% variance explained** | 32.243 | 23.604 | 19.931 |  | 63.500 | 34.400 |
| **Cumulative % explained** | 32.243 | 55.848 | 75.778 |  | 63.500 | 97.900 |
